# Left-sided compartmental resection of an 18 kg retroperitoneal liposarcoma: a case report

**DOI:** 10.1093/jscr/rjag671

**Published:** 2026-07-31

**Authors:** Prem Ghei, Sneha Balagopala, Saumitra Rawat, Anil Arora, Rajeev Malhotra, Sonia Badwal, Mithilesh Kumar, Abhishek Mitra

**Affiliations:** Institute of Surgical Gastroenterology, GI and HPB Onco-Surgery and Liver Transplantation, Sir Ganga Ram Hospital, Sir Ganga Ram Hospital Marg, Old Rajinder Nagar, New Delhi 110060, India; Institute of Surgical Gastroenterology, GI and HPB Onco-Surgery and Liver Transplantation, Sir Ganga Ram Hospital, Sir Ganga Ram Hospital Marg, Old Rajinder Nagar, New Delhi 110060, India; Institute of Surgical Gastroenterology, GI and HPB Onco-Surgery and Liver Transplantation, Sir Ganga Ram Hospital, Sir Ganga Ram Hospital Marg, Old Rajinder Nagar, New Delhi 110060, India; Institute of Liver, Gastroenterology, and Pancreaticobiliary Sciences, Sir Ganga Ram Hospital, Sir Ganga Ram Hospital Marg, Old Rajinder Nagar, New Delhi 110060, India; Department of Radiology, Sir Ganga Ram Hospital, Sir Ganga Ram Hospital Marg, Old Rajinder Nagar, New Delhi 110060, India; Department of Histopathology, Sir Ganga Ram Hospital, Sir Ganga Ram Hospital Marg, Old Rajinder Nagar, New Delhi 110060, India; Department of Anaesthesiology, Pain and Perioperative Medicine, Sir Ganga Ram Hospital, Sir Ganga Ram Hospital Marg, Old Rajinder Nagar, New Delhi 110060, India; Institute of Surgical Gastroenterology, GI and HPB Onco-Surgery and Liver Transplantation, Sir Ganga Ram Hospital, Sir Ganga Ram Hospital Marg, Old Rajinder Nagar, New Delhi 110060, India

**Keywords:** retroperitoneal tumour, liposarcoma, compartmental resection

## Abstract

Retroperitoneal liposarcomas are uncommon malignancies that often remain clinically silent and may attain massive size at diagnosis. Complete surgical excision with negative margins is the cornerstone of treatment; however, this is technically challenging due to indistinct anatomical planes and proximity to major vascular and visceral structures. Compartmental resection has been advocated to improve oncological outcomes, particularly with regard to local recurrence. We report a 43-year-old hypertensive male who presented with progressive abdominal distension and left-sided abdominal pain. Imaging demonstrated a large fat-containing retroperitoneal mass extending across the midline with multivisceral involvement. The patient underwent left-sided compartmental resection, including left nephrectomy, distal pancreaticosplenectomy, and left hemicolectomy. The resected specimen weighed 18 kg. Histopathology reported a dedifferentiated liposarcoma with negative margins. This case demonstrates the safety and feasibility of compartmental resection in achieving R0 resection in massive retroperitoneal liposarcoma.

## Introduction

Retroperitoneal tumours pose a significant surgical challenge, particularly when large or involving adjacent organs [1]. Liposarcoma is the most common histological subtype and often presents late due to its indolent growth and deep location, making differentiation between normal retroperitoneal fat and pathological tissue difficult [2]. Surgical resection remains the cornerstone of treatment; however, achieving clear margins is challenging due to ill-defined planes, inability to distinguish tumour from normal fat, and proximity to vital structures, resulting in high rates of margin positivity and local recurrence [3].

Compartmental resection, advocated by the TARPSWG (Trans-Atlantic Retroperitoneal Sarcoma Working Group), has been proposed to improve oncological outcomes through en bloc removal with adjacent organs and improved margin status [4, 5]. This is particularly relevant for large tumours or those involving critical structures. Reports of massive retroperitoneal liposarcomas treated with formal compartmental resection remain limited, especially from India. To our knowledge, no case describes a left-sided compartmental resection for a tumour of this magnitude.

## Case report

**Figure 1:**
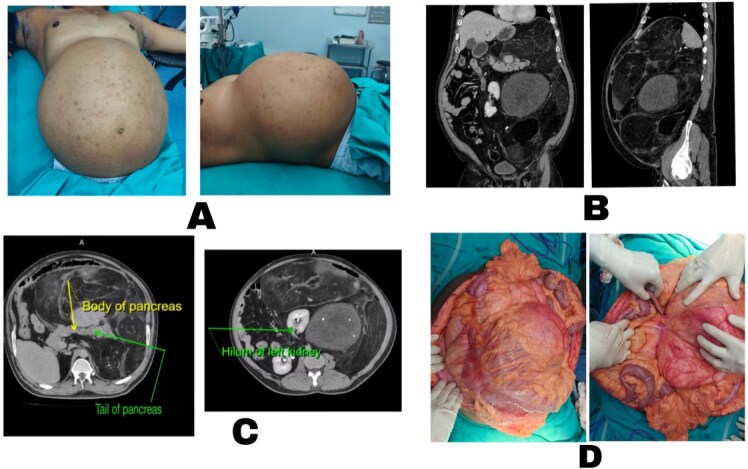
(A) Preoperative clinical photograph showing massive abdominal distension due to a large retroperitoneal tumour occupying the left abdomen and extending across the midline. (B) Coronal and sagittal section of tumour demonstrating the cranio-caudal extent of tumour. (C) The pancreas (body, tail, and neck) encased by the tumour. Left kidney and hilum also seen encased by the tumour. (D) Intraoperative photograph demonstrating a giant retroperitoneal lipomatous tumour occupying the abdominal cavity. Intraoperative view on medial rotation of the tumour with exposure of the root of the mesentery and medial tumour plane, enabling identification of major vascular structures during compartmental resection.

A 43-year-old hypertensive male presented with progressive abdominal distension and left-sided abdominal pain for 15 days. Pain was insidious, gradually worsening, and aggravated postprandially. There was no vomiting, altered bowel habits, weight loss, or gastrointestinal bleeding. Examination revealed a hemodynamically stable patient with a markedly distended abdomen and a large, firm, non-tender mass occupying most of the abdomen (Fig. 1A).

Ultrasonography showed a large heterogeneous lesion. Contrast-enhanced CT angiography revealed a massive retroperitoneal mass with predominant fat attenuation, thick septations, solid and myxoid components, and calcifications, extending from the left subphrenic region to the iliac fossa and crossing midline, displacing viscera to the right (Fig. 1B).

The tumour encased the pancreas (body and tail), left kidney, adrenal gland, and splenic, renal, and adrenal vessels (Fig. 1C). It abutted the aorta, inferior vena cava, and psoas muscle, with involvement of inferior mesenteric vessels and proximity to the superior mesenteric artery and celiac axis. These findings suggested dedifferentiated liposarcoma.

Following multidisciplinary discussion, a formal left-sided compartmental resection was planned. Through a midline laparotomy, a giant tumour occupying the left retroperitoneum was identified (Fig. 1D). A Cattell–Braasch manoeuvre exposed major vessels. The right ureter was identified and preserved. Major arterial structures were free of involvement.

**Figure 2:**
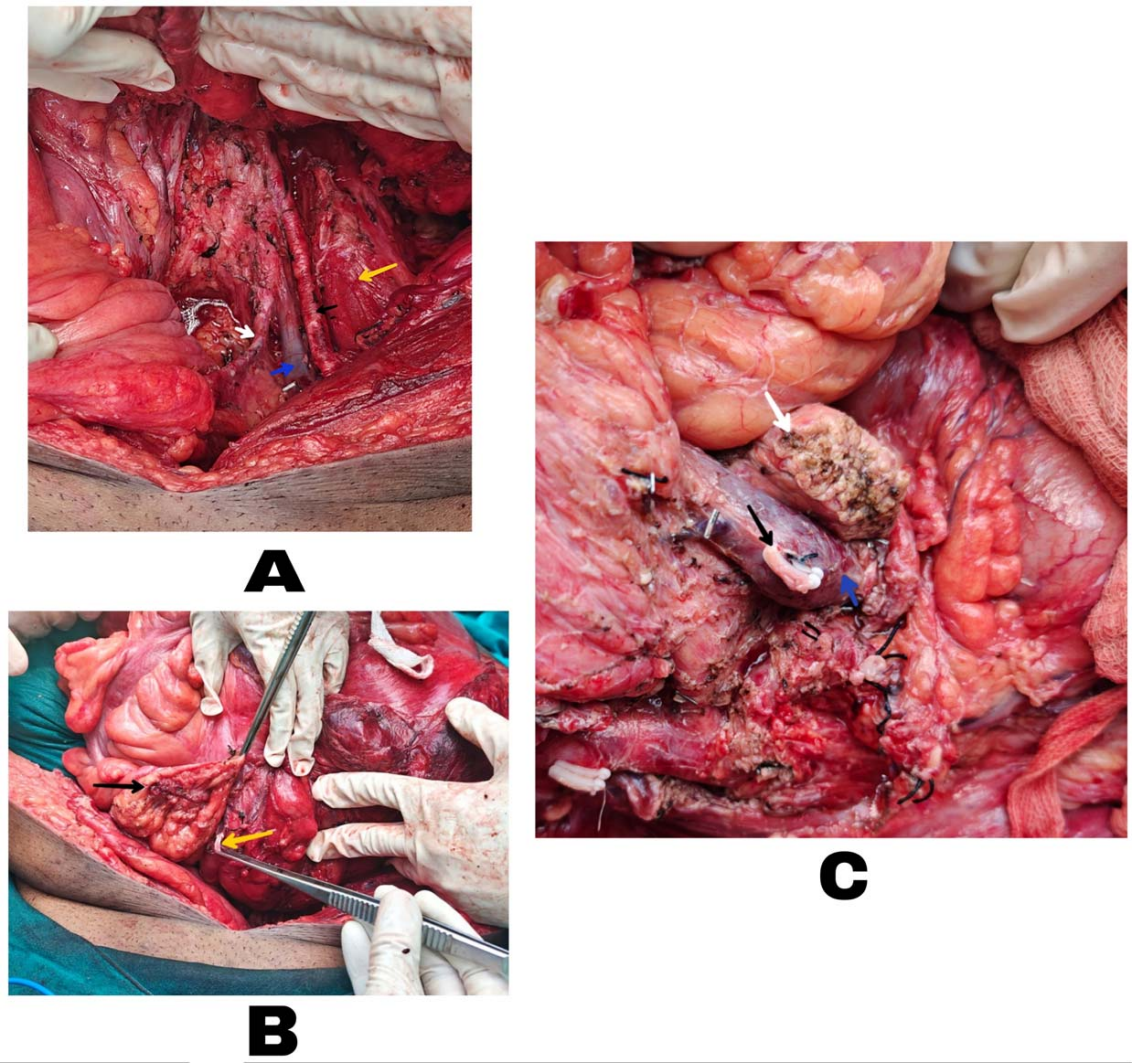
(A) Intraoperative photograph demonstrating the caudal-to-cranial approach during compartmental resection. The left psoas muscle is indicated by the yellow arrow, the left external iliac vein by the blue arrow, the left external iliac artery by the black arrow, and the internal iliac artery by the white arrow, illustrating exposure of major vascular structures during retroperitoneal dissection. (B) View from caudal end showed transected lower sigmoid colon marked with black arrow. Clipped left ureter marked with yellow arrow. (C) Intraoperative view following distal pancreatectomy with splenectomy. The splenic vein stump is indicated by the black arrow, the portal vein by the blue arrow, and the pancreatic stump by the white arrow, demonstrating the operative field after completion of pancreatic transection and vascular control.

A caudal-to-cranial and medial-to-lateral dissection was adopted to allow early vascular control. The peritoneum was dissected caudally and extended into the retroperitoneum. The left external iliac vessels were identified (Fig. 2A). The left ureter was clipped and divided. The inferior mesenteric artery was ligated at its origin to facilitate colon mobilization. Left renal and adrenal vessels were ligated.

The lesser sac was entered, duodenum kocherized, and stomach retracted. The inferior mesenteric vein was ligated. The rectosigmoid junction was mobilized and transected (Fig. 2B). Tumour involvement of distal pancreas and splenic vessels necessitated distal pancreatectomy and splenectomy. The splenic artery and vein were ligated at origin, the pancreas transected, and stump closed (Fig. 2C). The distal transverse colon was divided.

**Figure 3:**
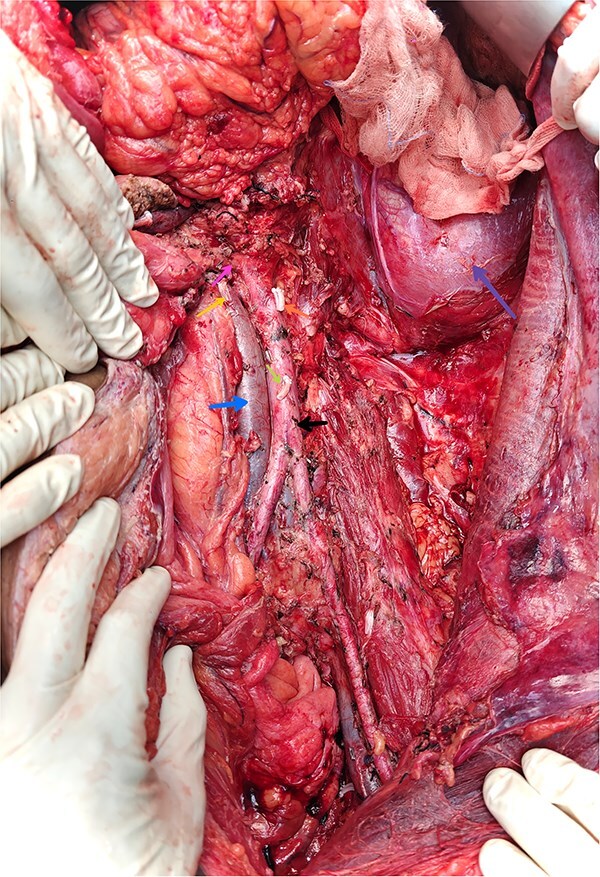
Tumour bed following left-sided compartmental retroperitoneal resection demonstrating major vascular landmarks. The SMA is indicated by the pink arrow, the IVC by the blue arrow, the left renal vein stump by the yellow arrow, the left renal artery by the orange arrow, the abdominal aorta by the black arrow, the IMA stump by the green arrow, and the left hemidiaphragm by the violet arrow. IMA, inferior mesenteric artery; IVC, inferior vena cava; SMA, superior mesenteric artery.

After vascular and visceral control, lateral and posterior dissection included en bloc resection of the left psoas fascia (Fig. 3 and Supplementary Video S1). The specimen was removed en bloc, including tumour, left kidney, adrenal gland, distal pancreas, spleen, hemicolon, and peritoneum. Due to intraoperative instability, bowel continuity was deferred and an end transverse colostomy fashioned. Abdomen was closed with drains in the tumour bed and near the pancreatic stump; blood loss was ~700 ml, with transfusion of 2 units pure red blood cells and 4 units fresh frozen plasma.

**Figure 4:**
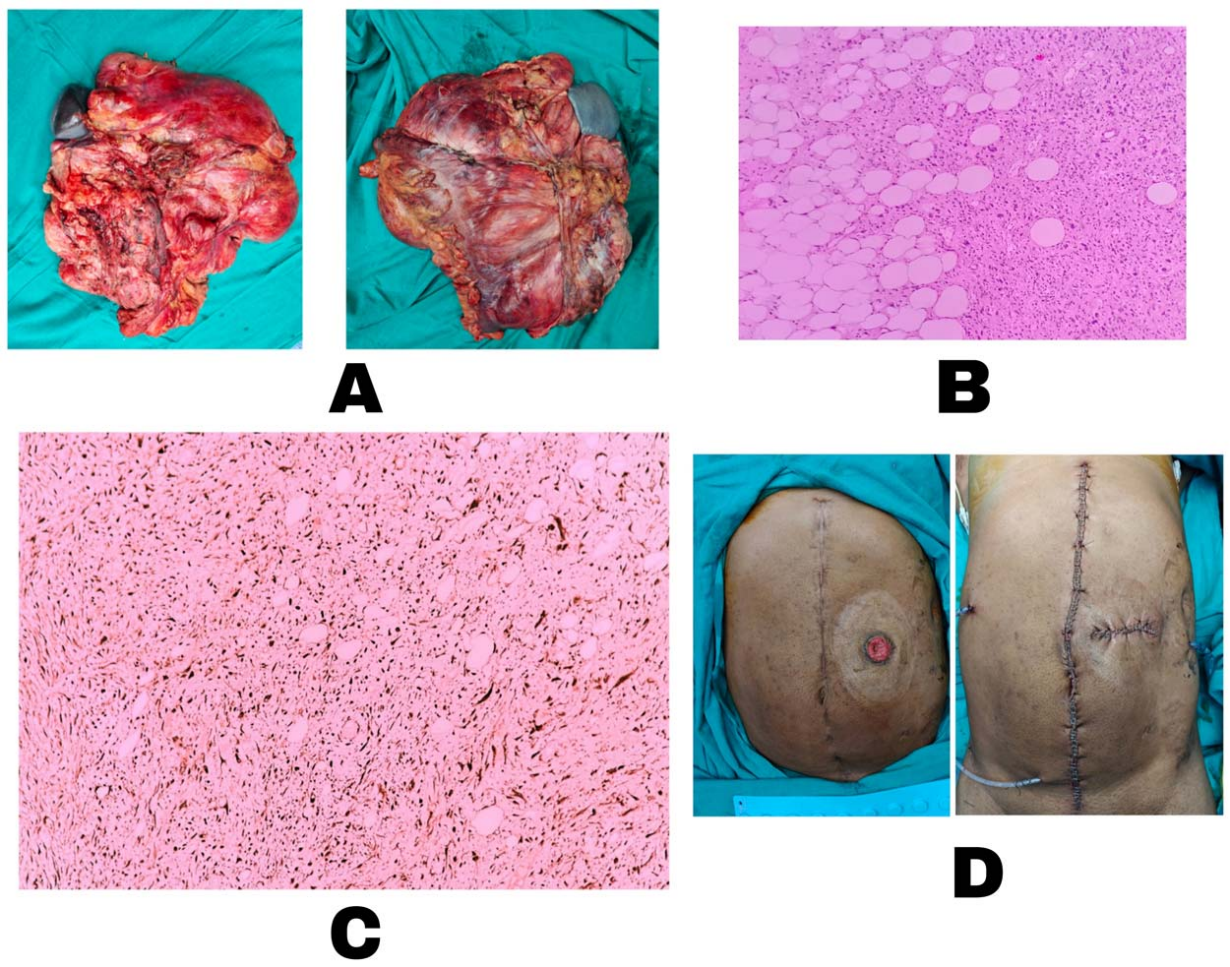
(A) Gross specimen including the retroperitoneal tumour along with the left kidney, adrenal gland, distal pancreas, spleen, left hemicolon, psoas fascia, and peritoneal layer. (B) Haematoxylin and eosin–stained section (×20 magnification) demonstrating a well-differentiated component of liposarcoma, characterized by proliferation of oval to spindle-shaped atypical stromal cells infiltrating and splaying between mature adipocytes. (C) Immunohistochemical staining for MDM2 (×20 magnification) demonstrating strong diffuse nuclear positivity in tumour cells, supporting the diagnosis of liposarcoma. (D) Preoperative and postoperative clinical images of the abdomen demonstrating the stoma prior to closure and the following Hartman’s reversal.

Gross examination showed a tumour measuring 60 × 40 × 16 cm and weighing 18 kg (Fig. 4A). Histopathology revealed dedifferentiated liposarcoma with transition from well-differentiated adipocytic areas to high-grade spindle cell sarcoma (Fig. 4B). Tumour showed nuclear atypia and necrosis. Immunohistochemistry was positive for MDM2 and CDK4 (Fig. 4C). Ki-67 was 10%–12% in well-differentiated and 50%–60% in dedifferentiated areas. The tumour infiltrated pancreas and encased adjacent organs, though these were histologically uninvolved. Margins were negative (R0). Surveillance was advised.

The patient recovered and was discharged on postoperative Day 14. A Grade B pancreatic fistula was managed conservatively. Drain removal occurred at 8 weeks. Hartmann’s reversal was performed at 3 months with no recurrence, and colorectal anastomosis was completed (Fig. 4D). Recovery was uneventful.

## Discussion

Complete resection with negative margins remains the most important prognostic factor in retroperitoneal sarcoma [1, 3]. Achieving R0 resection is challenging due to lack of defined planes and proximity to major structures. Difficulty in distinguishing tumour from normal fat further contributes to residual disease and recurrence [2, 4].

Large tumours often displace or encase organs and vessels, increasing operative complexity and risk of incomplete resection [6]. Conventional organ-preserving approaches are associated with higher positive margin rates [7].

Compartmental resection addresses these limitations by en bloc removal of tumour with adjacent organs within the involved compartment, irrespective of gross invasion [8, 9]. This TARPSWG-endorsed strategy prioritizes oncological clearance over organ preservation. Studies demonstrate improved local control and reduced recurrence, albeit with increased morbidity [5, 9].

Multivisceral resections involving kidney, colon, pancreas, and spleen are feasible in experienced centres with acceptable morbidity [10]. Long-term outcomes are primarily determined by margin status rather than organ preservation [11].

Giant liposarcomas >20 kg have been reported, though most underwent conventional rather than planned compartmental resection [12–15]. Reports of formal compartmental approaches in tumours of this magnitude remain limited. A step-wise intraoperative strategy—initial resectability assessment followed by caudal-to-cranial and medial-to-lateral dissection—minimizes vascular injury and improves completeness of resection.

This case demonstrates the feasibility of planned left-sided compartmental resection for an 18 kg retroperitoneal liposarcoma. Despite multivisceral involvement and vascular encasement, R0 resection was achieved, highlighting the oncological adequacy of compartmental surgery in massive retroperitoneal sarcomas.

## Supplementary Material

Liposarcoma_video_(1)_rjag671
